# Comparability of comparative toxicity: insect sensitivity to imidacloprid reveals huge variations across species but also within species

**DOI:** 10.1098/rspb.2023.2811

**Published:** 2024-06-12

**Authors:** Nicolas Nagloo, Elisa Rigosi, Lina Herbertsson, David C. O'Carroll

**Affiliations:** ^1^ Department of Biology, Lund University, Sölvegatan 35, Lund 22362, Sweden

**Keywords:** neonicotinoids, insect pollinators, bees, LD_50_, lethality, pesticides

## Abstract

Pesticides have been identified as major drivers of insect biodiversity loss. Thus, the study of their effects on non-pest insect species has attracted a lot of attention in recent decades. In general toxicology, the ‘gold standard’ to assess the toxicity of a substance is to measure mass-specific LD_50_ (i.e. median lethal dose per unit body mass). In entomology, reviews attempting to compare these data across all available studies are lacking. To fill this gap in knowledge, we performed a systematic review of the lethality of imidacloprid for adult insects. Imidacloprid is possibly the most extensively studied insecticide in recent times, yet we found that little is comparable across studies, owing to both methodological divergence and missing estimates of body mass. By accounting for body mass whenever possible, we show how imidacloprid sensitivity spans across an apparent range of approximately six orders of magnitude across insect species. Very high variability within species can also be observed owing to differences in exposure methods and observation time. We suggest that a more comparable and comprehensive approach has both biological and economic relevance. Ultimately, this would help to identify differences that could direct research towards preventing non-target species from being negatively affected.

## 1. Introduction

Assessing the toxicity of chemical compounds is fundamental to evaluating their potential risk. However, toxicity is a multidimensional concept which can be assessed at different endpoints (e.g. lethal versus sublethal effects), functional levels (e.g. whole organism, tissue, cellular, molecular and genetic levels) and time points (e.g. acute versus chronic exposure). At the same time, any of these can involve different exposure routes and application methods.

The most common and reductionistic toxicity endpoint is the assessment of lethality, i.e. whether an animal is killed by a specific dose of a compound. Independently of the compound and animal used, the gold standard approach has been to estimate a mass-specific LD_50_ (lethal dose 50): i.e. the dose (per unit body mass) that kills 50% of the population. This measure was first introduced by Trevan [1] in this very journal almost 100 years ago, who argued for the LD_50_ as a more robust measure than prior approaches to assess lethality and compare toxicity across compounds, organisms and conditions. Most commonly, LD_50_ is estimated by presenting different doses to different treatment groups of animals (often rodents) to build a so-called lethality curve that is then used to solve the mass-specific LD_50_ for the compound. This approach to toxicological testing has subsequently become mandatory for many chemical substances, including feed additives, pesticides and industrial chemicals, although subsequent work pointed out that LD_50_ is not always ethically justified because of the large number of sacrificed animals required [2]. Alternatives such as ‘up-and-down’ procedures may be useful to reduce the numbers of experimental animals required to estimate toxicity thresholds [3].

**Figure 1.**
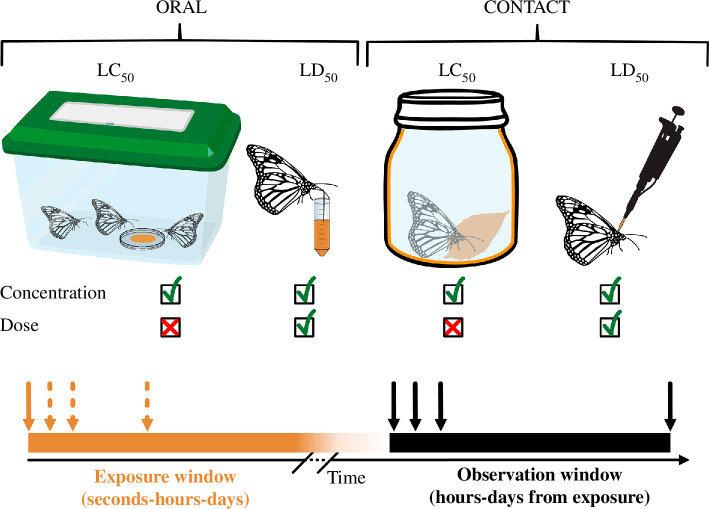
Schematic illustration of the main methods used to assess LC_50_s/LD_50_s of a pesticide in adult insects. Insects are exposed to a specific concentration of pesticide (orange) either via oral or contact administration. Independent of the administration route, the individual dose cannot be quantified if the volume of intake/contact per animal is not measured. The time of exposure and the time of observation after exposure varies across experimental methods from a few seconds to many days.

In entomology, lethality has also been extensively used to assess the toxicity of pesticides and evaluate their risks. For example, authorities such as the European Food Safety Authority (EFSA) require measures of lethality as one of the first steps for consideration of a newly formulated pesticide seeking approval for introduction to the market [4]. However, practical challenges in working with miniature animals, such as insects, mean that true LD_50_ standards such as those commonly applied in rodents have rarely been applied in entomology. More commonly, simpler metrics of mortality such as ‘LC_50_’, the median lethal exposure *concentration* (with an otherwise unknown individual dose) or the median lethal dose per animal (with unknown individual body mass) are more frequently used (figure 1). This begs the question of just how much is actually comparable across different studies and insect species for this assessment of toxicity?

Reviews attempting to compare toxicity data across all insect studies are scarce. Possibly the most comprehensive in recent times has been Hardstone & Scott [5], who compared the toxicity of 63 insecticides with topical application between the honeybee *Apis mellifera* and other insect species whose LD_50_s were known at that time. This work highlighted enormous differences in sensitivity (across approximately six orders of magnitude) among different insect taxa.

**Figure 2.**
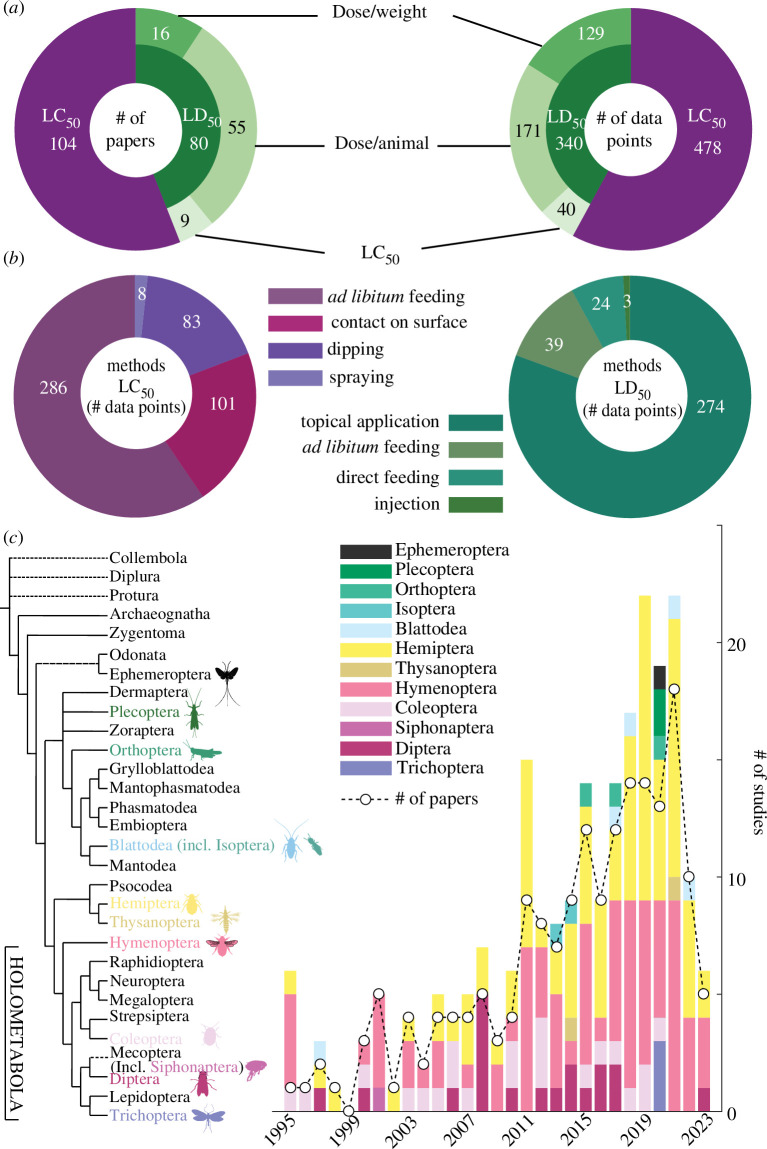
Descriptive summary of the extracted data. (*a*) Number of articles (left) and number of data points (right) used in our analysis (see §2 for a clarification of ‘data point’). For each of the two main categories, LC_50_ or LD_50_, we reported the methods of exposure used (*b*). (*c*) Number of articles published over the years superimposed to the number of studies represented per insect orders over the years (see §2 for the definition of ‘study’; insect phylogeny redrawn from [16], credits for some of the insect silhouettes goes to Melissa Broussard https://creativecommons.org/licenses/by/3.0/; Gareth Monger, https://creativecommons.org/licenses/by/3.0/; Didier Descouens,https://creativecommons.org/licenses/by-sa/3.0/; Gabriela Palomo-Munoz,https://creativecommons.org/licenses/by-nc/3.0/).

Here, we performed a systematic review of insects’ sensitivity to the neonicotinoid imidacloprid (IMI), possibly the most notorious and extensively studied insecticide in recent times [6]. Neurotoxic neonicotinoids are one of the best studied groups of pesticides and are commonly used worldwide [7]. These chemicals remain popular because of their specificity to insect nervous systems and thus their relative safety for humans and other vertebrates [8]. Older reviews have compared the range of neonicotinoid toxicity (lethal concentrations) across orders of aquatic invertebrates [9], bee species [10] or multiple studies of a single species—the widely studied honeybee, *A. mellifera* [11,12]. Owing, in part, to recent concerns for susceptibility of non-pest insects, and the potential role of pesticides in declining biodiversity [13–15], studies on neonicotinoids, and IMI in particular, have exploded in the decade since these earlier reviews (see figure 2).

Screening all possible studies that aimed to establish lethality to IMI, we found, to our surprise, that little is comparable across studies, owing to methodological divergence (figure 1) as well as missing estimates of body mass. For a subset of papers, we partially overcame this problem by applying reasonable literature-based assumptions for body mass. This approach allowed us to include data from 80 papers to compare the median lethal dose in ng/mg across more than 50 insect species. Our results confirm a huge apparent range (approximately six orders of magnitude) in IMI sensitivity across species. However, we also observed very high variability even within individual species, partly owing to differences in exposure methods and observation time.

## 2. Material and methods

### (a) Literature search

To capture all studies on IMI lethality conducted on adult insects, we used the Web of Science as our primary search engine, with the following query string:

((((TS=(‘imidacloprid’)) AND TS=(insect*)) AND TS=(‘survival’ or ‘toxicity’ or ‘mortality’ or *lethal*)) AND TS=(‘LD50’ or ‘LC50’ or ‘LD(50)’ or ‘dose’)).

We conducted this search initially on 14 February 2022, which yielded 596 results and formed the basis for our initial analysis. We subsequentially updated the search on 24 May 2023 to cover the most recent publications and obtained 72 new results. In addition, we added any papers we were aware of from our own databases if they did not appear in the results of the search in Web of Science (*n = 3*). Such omissions in papers picked up by our search query occur because lethality tests are not always the primary emphasis of the experiment and are therefore occasionally omitted from keywords, titles or abstracts. Instead, lethality trials may be used, for instance, to determine a *sublethal* dose where primary experiments aimed to study consequences of sublethal exposure.

### (b) Inclusion criteria and data extraction

We thoroughly examined each of the papers from our search results to ensure they met our minimum criteria of providing LC_50_ or LD_50_ data for adult insects exposed to IMI. We excluded studies conducted on sub-adult instars (including larvae of holometabolous species) or studies that lacked clear information on the methods and could not be allocated to a methodological category. In some cases, we found potential errors in reported data (e.g. inconsistencies in units between different figures) and we contacted the authors to confirm the correct value. In two cases, the authors did not reply, and the possible mistake was not obvious, so we excluded those papers and their data points from our dataset. In some cases, the reported LC_50_ or LD_50_ values were outside the limits of their reported 95% confidence interval. These values were excluded from the database.

For papers that met our inclusion criteria, we created one or more entries in our dataset. Simple studies of a single species, route of exposure and assessment timepoint (figure 1) generated a single database entry (‘data point’ or rows in the database). But often a single paper would also provide additional entries. For example, if a paper reported LD_50_s for two different species, with an LD_50_ calculated for both oral and topical exposure for each species, and with lethality assessed both at 24 and 48 h after exposure for all species and exposure methods, this would have led to eight entries. Studies on multiple populations and strains, if reported, were also treated as multiple entries (rows). Each of these entries is subsequently referred to as ‘data points’ in the text and figures. For each data point, we extracted: the year of publication; the name of the species; family; insect order; the strain used (if stated); any additional specific adult characteristics (e.g. sex, forager status and newly emerged); method/route of exposure (see figure 1); the IMI formulation used (e.g. commercial formula or pure compound/active ingredient); the duration of exposure and time of observation (see figure 1); the location sampled and the reported or calculated LC_50_/LD_50_ values with CIs (when reported). Our final dataset is available from the Swedish National Data Service [17].

### (c) LD_50_ estimation

For each recorded data point (i.e. either LC_50_ or LD_50_), if the mass-specific lethality was not given in the original article as dose/unit of body mass, we estimated it. In many papers, LD_50_ was given as dose/insect without specifying the body mass. In such cases, we searched the literature for alternative reports for the fresh body mass of adults of the same species. Where possible we matched the specific sex, caste or post-emergence age to that of the collected data point. In just one case [18], we could not find a reliable estimate of the fresh body mass. However, the authors reported the dry mass of the experimental animals in the paper. The values from this paper were included in our study but marked in electronic supplementary material, figure S1, with asterisks and in electronic supplementary material, figure S4, as open circles/diamonds, since the lower body weight results in a higher mass-specific LD_50_ compared with the mass-specific LD_50_ calculated for fresh body mass. In some additional cases where a study documented lethality as LC_50_ but specified a particular treatment volume (such as during topical application), we again calculated an estimated LD_50_ based on the specified concentration and the literature estimates of fresh body mass. We do not distinguish among different subspecies in figures (e.g. electronic supplementary material, figure S1 and S4) and analyses.

### (d) Comparing the effect of body mass across species

For data used to investigate the relationship between body mass and LD_50_ (§3e) we selected a single LD_50_ value for each species. To remove potential confounding effects from differences in methodology, we only selected representative data points from studies that used topical applications and a time of observation of 24–72 h. Typically, the lowest LD_50_ value reported for a species was selected. However, there were a few exceptions where we prioritized studies which used susceptible strains and technical grade IMI whenever possible. These exceptions include selections for *A. mellifera*, *Bombus terrestris* and *Piezodorus guildinii*. A linear regression model was fitted to the selected data using an iteratively re-weighted least squares method to reduce the effect of outliers on the model and an analysis of variance was used to test the linear regression model against a constant model. The analysis was done in Matlab (2022a, The MathWorks, USA).

### (e) Meta-analysis of differences among insect orders and exposure method

To quantify differences in estimated lethality among orders, we used R v. 4.2.3 and fitted a meta-analysis via linear models [19] with insect order as moderator. For this analysis, we only used studies that had reported CIs. We excluded studies where the LD_50_ was outside the reported CI and one study where the lower limit was reported as 0 and the upper was equal to the LD_50_. In total, we included 61 studies. We decided to include one study where LD_50_ was calculated per mg of body mass in dry weight. This study tested six species from three insect orders that were not covered by any other study. To meet the requirements of residual distribution, we selected log transformation when converting test statistics to sampling variances [19]. We predicted CIs [19] for the plots. Because the number of data points varied strongly among studies (1–62 per study), we reduced this variation by calculating a grand mean per species and study, instead of using a mixed-effect meta-analysis with the study as a random factor. For each study with more than one reported value per species, a grand mean CI was calculated per species using a meta-analysis via linear models. To evaluate differences in lethality depending on exposure method, we fitted a meta-analysis mixed-effects model [19]. In this analysis, we only included studies on *A. mellifera*, as this was the only species with multiple studies per exposure method. Eighteen studies on *A. mellifera* reported CIs with the number of data points reported per study ranging from 1 to 7. Six of the studies had used more than one method of exposure. We used a nested meta-analysis with study ID as random factor to account for the non-independence of LD_50_ values from the same study.

## 3. Results and discussion

### (a) Meta-analysis of diversity in applied methods

Our dataset generated data points from 184 articles (figure 2*a*; full reference list in the electronic supplementary material). Among these, the majority (57%, 104 articles) exclusively reported the LC_50_ and conducted experiments in a way that prevented us from estimating LD_50_ (figure 2*b*) primarily owing to a lack of standardization in pesticide exposure methods (figure 2*b*). Only 9% (16 articles) reported lethality as either median lethal dose/unit body mass or as dose/insect with a report of the average body mass that we could directly use in our analysis. In addition, we were able to use other literature to estimate the body mass and compute mass-specific LD_50_s based on 55 articles that reported LD_50_ as dose/animal and nine articles that reported LC_50_s but also applied a known volume. Thus, in total, we obtained 80 articles for which we had LD_50_s expressed as dose/unit of body mass.

The 184 articles in our dataset allowed us to extract a total of 818 data points (figure 2*a* right; see §2 for a clarification of our definition of a ‘data point’). The overall distribution of these data points is similar to that of the papers with a slightly higher proportion (16%) of mass-specific LD_50_. This higher proportion is mainly driven by a single article that reported LD_50_ in two planthopper species, with 41 different collection sites for one and 21 for the other, for a total of 62 data points. In total, we obtained 340 LD_50_ data points from 80 papers.

The entire dataset (184 papers and 818 data points) contains 115 species (ignoring subspecies, study-specific strains and taking into account that synonyms such as *A. citricola* and ***A. spiraecola***; *Homalodisca coagulate* and ***H.*** ***vitripennis*** refer to the same species, species names in bold were selected for these two synonyms). These 115 species span across 12 insect orders (figure 2*c*). Mass-specific LD_50_ data were found for 52 species across 11 of these orders, with the exception being Thysanoptera (thrips). The earliest papers date from 1995. If we define a study as a single species within a unique article (i.e. a paper that tested three species would count as three studies) both the number of papers and the number of studies per year increased dramatically from the 1990s to 2021.

### (b) IMI sensitivity spans across approximately six orders of magnitude

**Figure 3.**
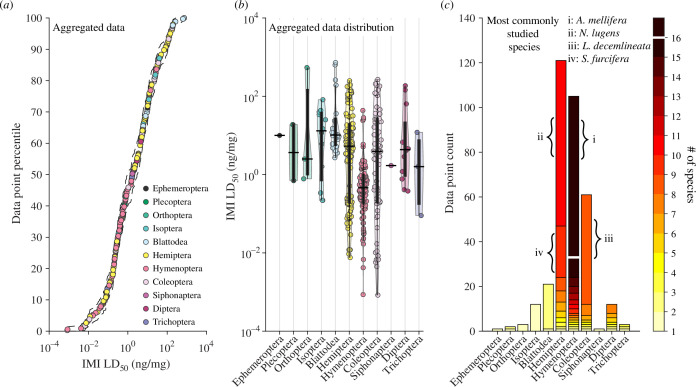
The distribution of LD_50_ estimates in our dataset across insect orders. Estimated LD_50_ values of IMI in ng per mg of body mass vary across approximately six orders of magnitude in adult insects. (*a*) An empirical cumulative distribution function of our aggregated data with 95% CI (dashed lines), (*b*) data distribution within each of the 11 insect orders for which LD_50_ estimations in ng/mg were possible, (*c*) The number of data points and species in each insect order showing how different insect orders are represented in the literature. Coloured and filled circles in panel (*a*) and (*b*) denote single LD_50_ estimates, with colours representing insect order as shown in the panel (*a*) figure legend. The black horizontal bars in panel (*b*) indicate the median LD_50_ of each insect order while the elongated black boxes under it denote the interquartile range. For the y-axis of panel (b), a base-10 log scale is used. Shaded contours in panel (*b*) indicate the density of LD_50_ measures within insect orders. The colour bar in panel (*c*) shows the number of species in each insect order.

When all the data are aggregated, our estimated body mass-corrected LD_50_s of IMI for 52 species of adult insects span across a staggering range of approximately six orders of magnitude (figure 3*a*; electronic supplementary material, figure S1). The lowest LD_50_ value is found in the Colorado potato beetle (Coleoptera, *Leptinotarsa decemlineata*; susceptible field population: 0.00084 ng/mg [20]) while the highest value is found in the German cockroach (Blattodea: *Blattella germanica*; resistant strain: 723.24 ng/mg [21]). Of the 11 orders represented, Hymenopterans and Hemipterans provide the highest number of data points with 105 and 121 data points, respectively. Some orders such as Ephemeroptera (*Ephemera danica*) and Siphonaptera (*Ctenocephalides felis*) are only represented by a single species and data point. The orders with the most data points tend to be the most speciose with 16 species of Hymenopterans, 10 species of Hemipterans and eight species of Coleopterans (figure 3*c*).

### (c) Variation in IMI sensitivity across insect orders

The median LD_50_ across the 11 insect orders studied to date range from 0.41 ng/mg in Hymenopterans to 13.23 ng/mg in Isopterans (figure 3*b*). LD_50_ in most insect orders cluster around the median in what appears to be a unimodal distribution. In contrast, Hemipteran LD_50_ values appear to be almost bimodal (figure 3*a,b*). Note, however, that this distribution is dominated by a high number of measures from two species (upper mode: *Nilaparvata lugens*, lower mode: *Sogatella furcifera*, electronic supplementary material, figure S1). While the meta-analysis indeed verifies that the LD_50_ values differ among insect orders (*Q*_M_ = 31.85, d.f. = 10, *p* = 0.0004; electronic supplementary material, figure S4), perhaps the most striking feature of the sensitivity distributions across different insect orders is their degree of overlap. The enormous spread of studies within insect orders, or even species, results in large residual heterogeneity (*I* ^2^ = 99.35%, *Q*_E_ = 9425.08, d.f. = 69, *p* < 0.0001; electronic supplementary material, figure S4) in the samples sizes among these distributions. Nevertheless, some features of these distributions deserve further discussion. In particular, the upper quartile in the Hymenopteran distribution (at 0.90 ng/mg) is lower than the median LD_50_ of all other insect orders. Most of the Hymenopteran data points come from *A. mellifera* which accounts for most of the variation within the order. *A. mellifera* LD_50_ values range from 0.0009 ng/mg to 5.93 ng/mg, thus spanning across 3.84 orders of magnitude in a single species. The most IMI tolerant Hymenopteran is the parasitoid wasp *N. vitripennis* (14.26 ng/mg), a value which is almost two orders of magnitude lower than the most tolerant pest species (*B. germanica*, 723.24 ng/mg). Taken together, these observations suggest that this insect order is intrinsically more sensitive to IMI than other insect orders studied to date.

### (d) Distribution of data points across methodologies

**Figure 4.**
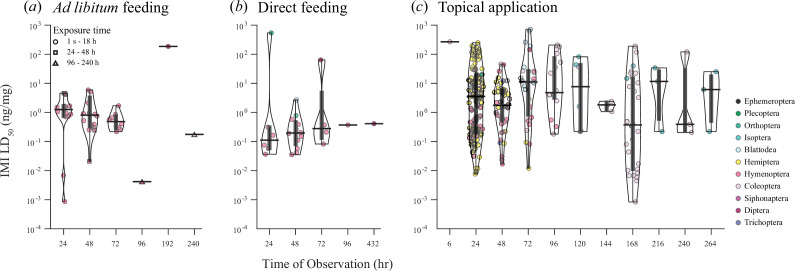
The distribution of LD_50_ values across time of observation of mortality ranging from 6 to 432 h and across exposure methods which include *ad libitum* feeding (*a*), direct feeding (*b*) and topical application (*c*). Coloured and filled circles represent single data points of LD_50_ measures estimated from literature. The colour of the circles represents insect order as detailed in panel (*c*). For all y-axes in panel (*a*), (*b*) and (*c*), a base-10 log scale is used. , a base-10 log scale is used. The black lines indicate the median LD_50_ of each group while the black box under it indicates the interquartile range. The contour of the violin plots indicates the density of LD_50_ measures. Exposure times across dataset were divided into three groups: 1 s–18 h (circles), 24–48 h (squares) and 96–240 h (triangles).

Figure 4 shows LD_50_ data broken down by observation time for three different methods of exposure: *ad libitum* feeding, direct feeding and topical application (figure 4). *Ad libitum* feeding exposes the insect to the pesticide through oral and contact routes, often for prolonged periods. Direct feeding exposes the insect through oral routes only, typically for brief exposure periods. Topical application exposes the insect through direct contact only for very brief exposure periods. Irrespective of the exposure method, observation time varied enormously across the dataset, although most studies used observation times of 24–72 h. Median LD_50_ values do not obviously decrease at longer times of observations. This suggests that it is other factors, such as insect order, species and the exposure time to the pesticide that primarily affect LD_50_ measures. Indeed, Hymenoptera are over-represented in both the *ad libitum* and direct feeding data (representing 92.5% and 80% of data points, respectively). By comparison with these oral exposure routes, topical application is more widely used for toxicology assays. Only 18.8% of data points for this exposure route are from Hymenoptera.

### (e) Effect of body mass on our perception of species sensitivity

**Figure 5.**
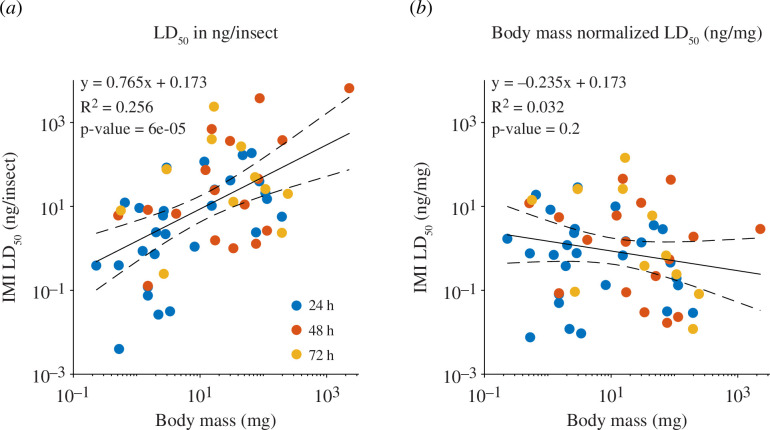
Bigger insects are not more tolerant to IMI when their body mass is taken into account. The relationship between insect body mass and their IMI toxicity when their LD_50_ is expressed either in ng of IMI per insect (*a*) or in ng of IMI per unit of bodyweight (*b*). For the y-axes of panel (*a*) and (*b*), a base-10 log scale is used. Solid lines in panel (*a*) and (*b*) represent the linear regression model fitted to the data, and the dashed lines represent the 95% CI of the model. The *p*-value provided in both panels indicates whether the models are significantly different from a constant (null) model. Single data points are selected per species for studies reporting topical applications only (see §2 for details on the criteria).

A widely held view, debated in recent regulatory guidelines [22], is that with increased body mass, insects become less sensitive to pesticides. However, data systematically exploring the relationship between body mass and pesticide toxicity across species is lacking. In figure 5*a*, we show that there is indeed a significant linear relationship between body mass and pesticide toxicity reported as a dose per animal (data selected per species, topical application only, see §2). This builds on previous findings of a similar relationship between body mass and IMI toxicity found in bees [22] and extends it across insect orders. However, when we accounted for the mass-specific toxicity by using LD_50_ reported in ng/mg, this trend possibly reverses direction, i.e. larger insects are, if anything, more sensitive per unit of body mass (figure 5*b*). While not statistically significant in the data aggregated across all orders (figure 5*b*; *r* ^2^ = 0.032, *p* = 0.2), this weak negative trend is largely owing to data from Hymenoptera, in which there is a significant negative pattern (*r* ^2^ = 0.29, *p* = 0.01; electronic supplementary material, figure S2). This suggests that other factors, including phylogenetic traits, are a better explanation for the differences in sensitivity across species. Modelling of toxicokinetics and toxicodynamics in aquatic vertebrates suggests that while body mass might be a good predictor of sensitivity within species, other parameters of the model account for differences between species [23].

Regardless of the explanation for the residual variance, our analysis underscores the significant effect of body mass as a potential confounding factor in studies which aim to track changes in sensitivity to pesticides or compare sensitivity across species. One commonly used approach is the Species Sensitivity Distribution (SSD) model, widely used to assess which species are most vulnerable to a novel pesticide. The SSD relies on toxicity data ranked across species, regardless of their body mass. However, accounting for the effect of body mass across species can lead to changes in rank of up to 55 percentile points for individual species (electronic supplementary material, figure S3). To partially overcome the confounding effect of insect body mass, new documentation published by *EFSA* [22], suggests that insect body mass should be reported when toxicological assays are conducted. While this is a step in the right direction, most recent studies are still far below standards originally adopted by Trevan [1] when the LD_50_ was first introduced.

### (f) What can the model species *A. mellifera* tell us about intra species variability across toxicity studies?

**Figure 6.**
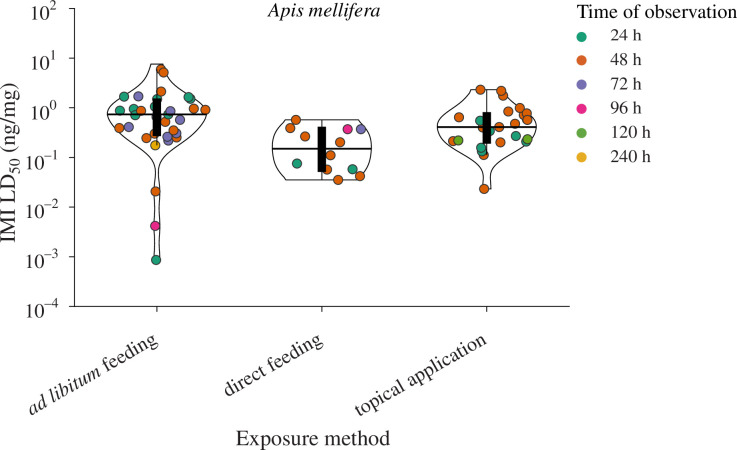
LD_50_ estimates for *A. mellifera* and their distribution across methodologies show the highest toxicity in direct feeding and the lowest in *ad libitum* feeding. A base-10 log scale is used for the y-axis. Colours indicate the time of observation of mortality during experiments as illustrated in the legend. Horizontal black lines represent the median LD_50_ of each distribution while the black box underneath indicates the interquartile range. The contours of the violin plots illustrate the density of data points.

The western honeybee, *A. mellifera*, is represented here by 71 estimates of the LD_50_ value based on data extracted from 22 independent studies. LD_50_ values ranged from 0.0009 ng/mg to 5.93 ng/mg spanning over 3.84 orders of magnitude within the species (figure 6; electronic supplementary material, figure S5). Out of the three main methods used to expose honeybees to IMI, *ad libitum* feeding results in the biggest spread of LD_50_ estimates, providing both the lowest and highest LD_50_ values. This can be partially attributed to variability in experiment duration, with bees observed for more than 96 h in many *ad libitum* toxicological assays. For shorter observation times (24–72 h) LD_50_ values separate along methodologies (electronic supplementary material, figure S5; meta-analysis: *Q*_M_ = 1718, d.f. = 2, *p* < 0.0001, although the predictions should be carefully interpreted owing to large heterogeneity: *Q*_E_ = 2264, d.f. = 36, *p* < 0.0001, these results were robust to the removal of two residual outliers). Direct feeding is associated with a greater sensitivity to IMI than topical application or *ad libitum* feeding (figure 6; electronic supplementary material, figure S5, and S6). While a difference between oral and topical application routes is not entirely surprising, the lower apparent sensitivity with *ad libitum* feeding is more difficult to explain, since this is also primarily an oral exposure route. One possibility is that with *ad libitum* access, bees within a treated group may consume highly variable amounts before intoxication. Those who consume far more than a lethal dose would then bias the overall estimated effect of that concentration. The difficulty in determining the exact intake by each individual as well as the timing of this intake relative to the observation time, are likely also contributing to the large variability among LD_50_ values with *ad libitum* feeding, which is the method with most variation also when limiting observation time to a maximum of 72 h.

### (g) Incomparability across species and methodologies

Our study compared the lethality of IMI, one of the most extensively investigated pesticide, with adult insects across all species that have been studied to date (up to May 2023).

Our knowledge, just one review, in 2010, has attempted to reconcile insecticide toxicity across insect species using LD_50_ values in dose/body mass. Hardstone & Scott [5] compared the topical toxicity of 63 insecticides between *A. mellifera* and species whose LD_50_s in µg/g were known at that time, including IMI, for which corresponding LD_50_ was available for 19 species. However, the authors did not take into consideration data from LC_50_ studies, oral applications nor discussed any possible factors that could explain variability (e.g. time of observation and purity of the pesticide).

Our dataset represents data for almost three times the number of species than this older review, probably reflecting the increased interest in the topic over the last decade [7]. However, our analysis also highlights how little is comparable across studies. First, the vast majority of studies reported toxicity as LC_50_ rather than LD_50_ owing to a lack of standardization in treatment methodologies. This actively and directly leads to incomparability across studies, resulting in the loss of the majority of the data we collected. Second, altogether the dataset covers a limited number of species, primarily from the Hymenoptera and Hemiptera orders, mainly represented by a selected number of pollinators and pests. Although this selection bias is an understandable consequence of the need to study toxicity in economically important species, it also underscores the fact that the sensitivity to IMI remains entirely unknown for most insect orders.

### (h) IMI tolerance: examples from our dataset

Our dataset comprises 340 individual estimates of body mass corrected LD_50_ values which span across approximately six orders of magnitude. The majority of these data points (250 of 340) are from just 6 of the 52 total species represented. These six species are either pest species (*N. lugens*, *n *= 74; *L. decemlineata*, *n *= 49; *S. furcifera*, *n *= 23; *B. germanica*, *n *= 20; *Reticulitermes flavipes*, *n *= 12) that have significant economic impacts or beneficial insects (*A. mellifera*, *n *= 71) that are agriculturally important.

The observed spread of IMI toxicity both within and across insect orders suggests that our ability to predict how diverse insect species will respond to IMI and other pesticides is severely limited. Despite this, by examining these six species we can explore how variation in exposure methods, animal strain and insect geographic origin has an impact on properties such as acquired resistance versus innate sensitivity. Exposure to IMI across multiple generations has produced resistant strains in multiple insect species. IMI tolerance is a major source of variation in our dataset, with examples of resistance gradually acquired in the field and in the laboratory [20,24–26]. In planthoppers (*N. lugens* and *S. furcifera*), sampling wild populations over time has shown that LD_50_ values can change significantly within 2 years of exposure to IMI in rice plantations [24]. For termites (*R. flavipes*), contemporary samples revealed differences in the sensitivity of termites to IMI between Louisiana and Maryland in the United States [27]. In contrast to these field-collected specimens, the highest LD_50_ value in our dataset comes from the German cockroach, *B. germanica*, where resistant strains were created by cockroaches while exposing them to IMI at the initial LD_25_ for this species for a single generation [28].

### (i) Mechanisms for IMI tolerance

The two main mechanisms which mediate IMI tolerance are (i) target site mutations of nicotinic acetylcholine receptor (nAchR) subunits which reduce their binding affinity for IMI and (ii) enhanced metabolism of IMI through the over-expression of detoxifying enzymes such as P450 monooxygenases, ATP-binding cassette transporters and gluthatione-S-transferase, among others [29–31]. Although target site mutations can produce up to a 250-fold increase in resistance to IMI, as observed in the brown planthopper, *N. lugens*, enhanced resistance of this magnitude is generally not found in wild populations as the fitness cost of such a mutation (Y151S) is too high [32–34]. The first and only comparable mutation observed in the field is from the green peach aphid, *Myzus persicae* found in France and Spain, which gained an approximately 234-fold resistance through the R81T mutation by greatly reducing the binding affinity of the β1 nAchR subunit to IMI [35].

On an evolutionary time scale, differences in the expression of P450 cytochromes may also explain the increased sensitivity to IMI that we noted earlier in the Hymenoptera (see §3c). Previous studies have revealed that the P450 cytochromes expressed by honeybees and bumblebees (CYP9Q3 and CYP9Q4, respectively) are not very efficient at metabolising IMI [36]. This may be partially owing to the metabolites being more toxic to the honeybee than the parent compound [37]. While phylogenomic and functional characterization has revealed some degree of conservation of CYP9Q-related genes across Hymenoptera, even these are not universally expressed, having been lost in some Megachilidae, for example [38]. Taken together with the extreme variation in IMI sensitivity we observe across other orders, this underscores the importance of more careful consideration of the effects of pesticides on diverse species.

### (j) How useful are the OECD guidelines for toxicity assessment?

Rising concerns about an accurate assessment of pesticide toxicity and its impact on important bee pollinators have led to the creation and adoption of internationally recognized guidelines for toxicity bioassays for honeybees and bumblebees, published by the Organisation for Economic Co-operation and Development (OECD) [39–43]. While similar concerns for non-bee species exist, no other guidelines have been recognized and adopted as widely as the ones developed for bees. Despite being species specific, many criteria between the guidelines of honeybees and bumblebees overlap for acute toxicity assays and are broadly applicable to non-bee insects. These include reporting the LD_50_ in µg per insect, the use of vehicle controls, an exposure window of ≤4 h, the correction of mortality and how to adjust the time of observation depending on insect mortality throughout the experiment. However, in the literature, we find that most acute pesticide toxicity studies simply report the LC_50_ and are unable to appropriately quantify individual exposure. The lack of individual monitoring in these studies makes it impossible to exclude non-feeding individuals (described in [43]) and leads to the underestimation of species sensitivity to pesticides such as IMI.

### (k) Key recommendations for future work

Our results highlight the limit of reporting LC_50_ rather than LD_50_ data; in 57% of articles analysed, the parameter used to report lethality was expressed in concentration rather than dose. We strongly argue that we should move away from LC_50_ studies and journals should not accept this parameter alone to support lethality, unless it is strictly necessary, e.g. in aquatic environments. Even in an aquatic environment, chemical analysis revealing the amount of pesticide in the body might be crucial to correlate the dose intake to the reported effects. Following the OECD guidelines (i.e. at least reporting body mass) increases the comparability; however, we argue that studies should report body mass corrected LD_50_ values (i.e. dose per unit body mass). Currently, only 9% of the literature reviewed here reported such values. Furthermore, group feeding compared with individual feeding makes it impossible to correctly determine the individual intake and if this varies among individuals, this method will result in an inaccurate estimation of LD_50_. As such toxicity datasets grow, meta-analysis in future work may provide important insights into such impacts. Our meta-analysis was often frustrated by the lack of CIs for reported LD_50_ values, so we would strongly encourage future workers to use methods that permit reporting of such values. Finally, our search revealed that toxicity (lethality) data are sometimes hidden in the nested design of the study. We strongly recommend the authors to indicate that they have estimated the LD_50_ in the abstract or keywords, even when lethality is only the first step to test sublethal effects of a pesticide.

Not only is a more comparable approach needed but also a more comprehensive one. Expanding the number of orders of insects studied, for example, could be crucial to judge probably impacts on diverse ecosystems. A more inclusive approach in the study of effects of pesticides on non-target insect is of crucial importance and could have both economic and ecological value. Finding species that show striking differences in sensitivities could spark new research into the mechanism behind these differences and allow us to identify new compounds that could act more or less selectively on a range of insect species sharing the same environment.

## Data Availability

Data are available from the Swedish National Data Service [17]. Supplementary material is available online [44].
